# Crystal structure and Hirshfeld surface analysis of 2-oxo-4-phenyl-2,5,6,7,8,9-hexa­hydro-1*H*-cyclohepta­[*b*]pyridine-3-carbo­nitrile

**DOI:** 10.1107/S2056989025010771

**Published:** 2026-01-01

**Authors:** Uthirapathi Rajapandiyan, Muruganandham Rajkumar, Haridoss Manikandan, Velusamy Rajathi, Sivashanmugam Selvanayagam

**Affiliations:** a Department of Chemistry Annamalai University, Annamalainagar Chidambaram 608 002 India; b PG & Research Department of Zoology, Government Arts College, C Mutlur, Chidambaram 608 102, India; c PG & Research Department of Physics, Government Arts College, Melur 625 106, India; Vienna University of Technology, Austria

**Keywords:** cyclo­heptene derivatives, inter­molecular hydrogen bonds, Hirshfeld surface analysis, crystal structure

## Abstract

The two mol­ecules in the asymmetric unit of the title compound, C_17_H_16_N_2_O, have a structural overlap with a root-mean-square deviation of 1.11 Å. Both seven-membered cyclo­heptene rings adopt a chair conformation. Reciprocal inter­molecular N—H⋯O hydrogen bonds between neighbouring mol­ecules lead to the consolidation of their mol­ecular conformations. Weak C—H⋯π inter­actions between neighbouring mol­ecules are also present in the crystal. The inter­molecular inter­actions were qu­anti­fied and analysed using Hirshfeld surface analysis, revealing that H⋯H inter­actions contribute the most to the crystal packing (45.4%).

## Abstract 2

The two mol­ecules in the asymmetric unit of the title compound, C_17_H_16_N_2_O, have a structural overlap with a root-mean-square deviation of 1.11 Å.

## 1. Chemical context

The core structure of the title compound contains several pharmacophores that are known for various biological activities: the seven-membered cyclo­heptene ring enhances lipophilicity and flexibility, features that are also found in cyclo­hepta­pyridine and azepine derivatives, which have shown strong anti­cancer and anti­microbial effects (Belal, 2014 ▸). The pyridine moiety is present in several FDA-approved drugs such as nicotinamide and isoniazid, both known for their anti­microbial and anti-inflammatory activities (De *et al.*, 2022 ▸; Mohamed *et al.*, 2021 ▸). The 2-oxo (pyridone) moiety, common in compounds like leflunomide and tenofovir, contributes to enzyme inhibition, anti­viral, and anti­cancer properties through hydrogen-bonding inter­actions with biological targets (Das & Sengupta, 2025 ▸). The cyano (—C≡N) group is often found in nitrile-based kinase inhibitors and anti­cancer agents, improving receptor affinity and metabolic stability (Fares *et al.*, 2021 ▸). The phenyl ring aids in π–π stacking and hydro­phobic inter­actions, enhancing cell permeability and binding efficiency, similar to that observed in quinoline and benzo­pyridine analogues (Zhu *et al.*, 2021 ▸; Rajapandiyan *et al.*, 2025 ▸). The combination of these functional groups creates a synergistic framework capable of multiple biological inter­actions. Therefore, the title compound and its structural analogues hold significant promise as multi-target therapeutic leads with potential anti­cancer, anti­microbial, and enzyme-inhibitory activities.

In the present work, the crystal structure and Hirshfeld surface analysis of the title compound, (I), are reported.
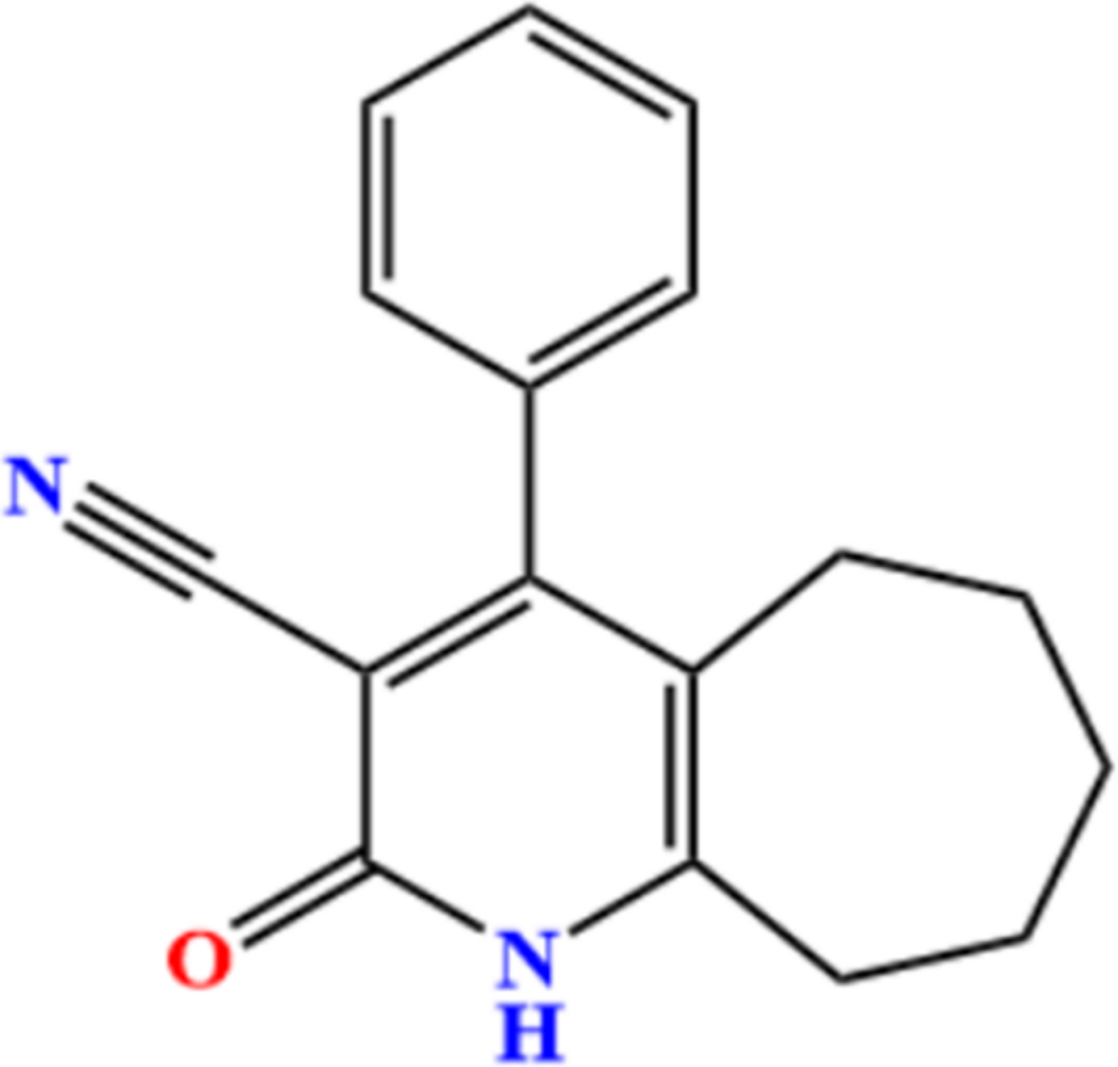


## 2. Structural commentary

**Figure 1:**
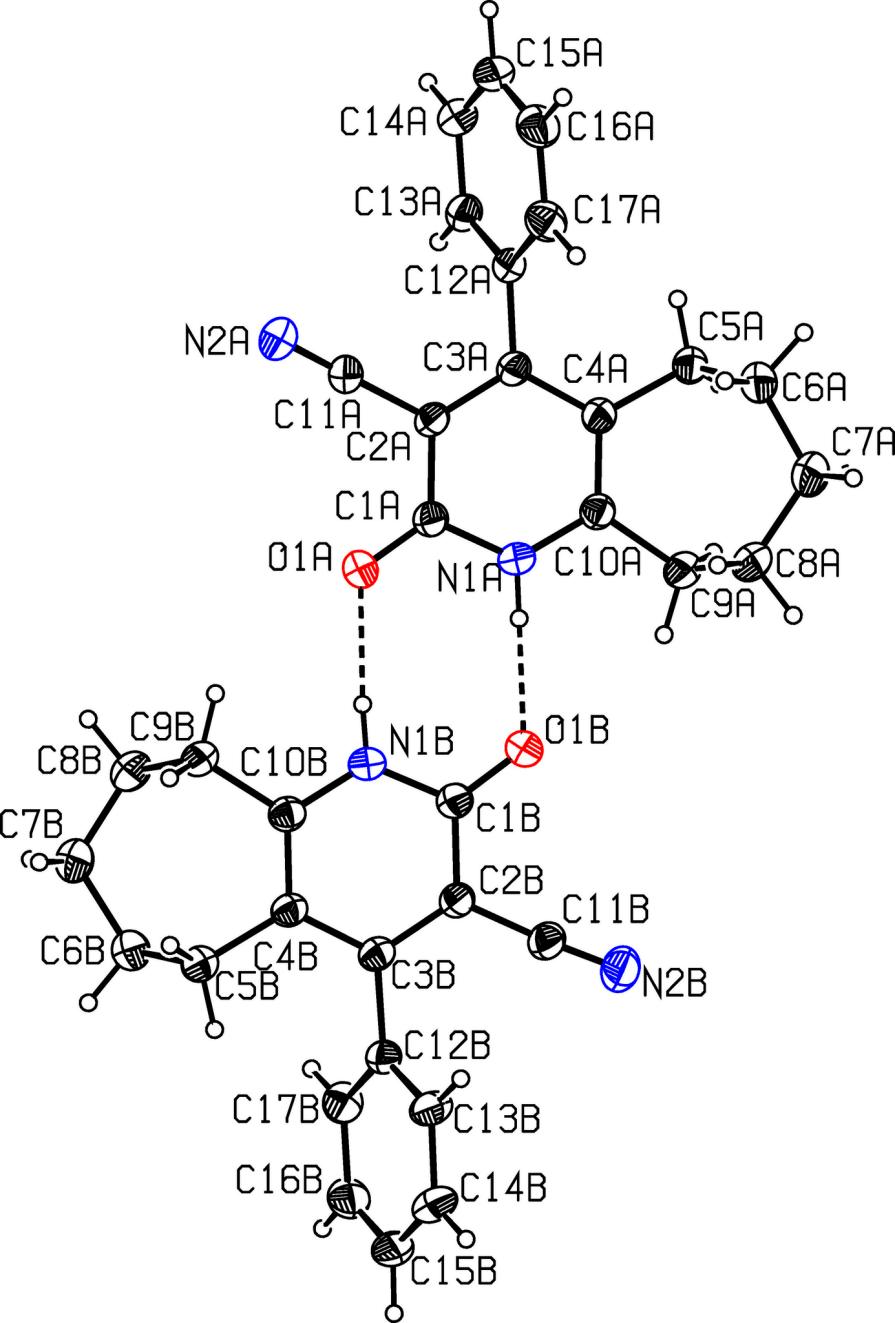
The two mol­ecules in the asymmetric unit of compound (I), showing the atom labelling. Displacement ellipsoids are drawn at the 30% probability level. Inter­molecular hydrogen bonds are shown as dashed lines.

**Figure 2:**
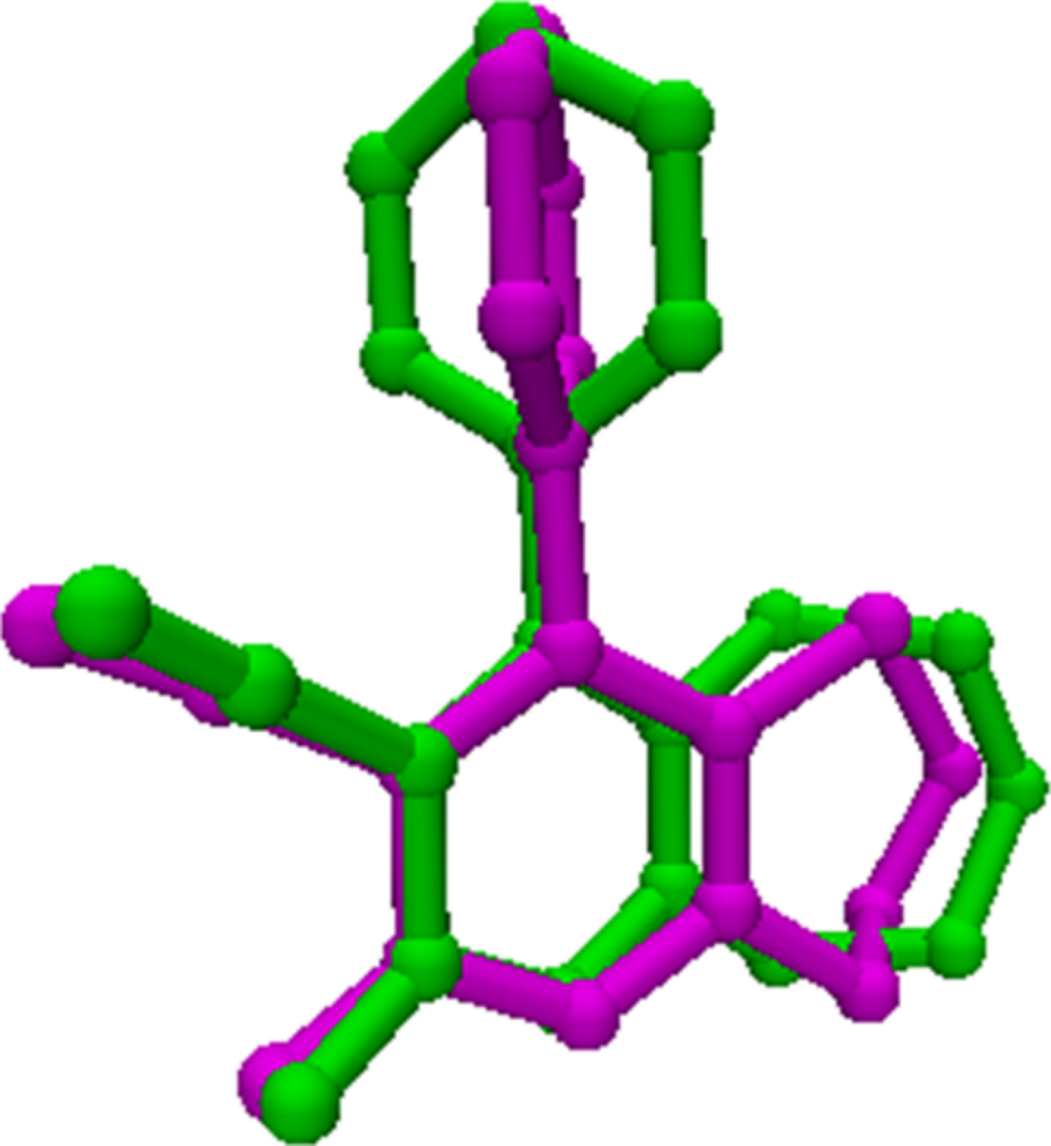
Superposition of mol­ecule *A* (green) and mol­ecule *B* (pink) in compound (I).

There are two mol­ecules in the asymmetric unit, *A* and *B* (Fig. 1 ▸). Fig. 2 ▸ shows a superposition of the two mol­ecules using *Qmol* (Gans & Shalloway, 2001 ▸); the root-mean-square deviation is 1.11 Å. The observed deviation is attributed to the torsional twisting of the phenyl ring with respect to the pyridine ring. For example, the torsion angle between atoms C17—C12—C3—C2 is 109.5 (5)° in mol­ecule *A* and −115.4 (5)° in mol­ecule *B*. The bond lengths N2*A*—C11*A* [1.135 (7) Å] and N2*B*—C11*B* [1.145 (6) Å] confirm the triple-bond character. The seven-membered cyclo­heptene ring (C4–C10) in both mol­ecules has a chair conformation, with puckering parameters (Boessenkool & Boeyens, 1980 ▸) *q*_2_ = 0.438 (5) and *q*_3_ = 0.627 (6) Å in mol­ecule *A* and *q*_2_ = 0.435 (4) and *q*_3_ = 0.648 (5) Å in mol­ecule *B*. Atoms C4*A*, C10*A* and C7*A* deviate by 1.075 (4), 1.046 (4) and −0.629 (6) Å, respectively, from the least-squares plane through the remaining four atoms (C5*A*/C6*A*/C8*A*/C9*A*) of the ring in mol­ecule *A*. The corresponding deviations in mol­ecule *B*, are −1.094 (4), −1.059 (4) and 0.656 (5) Å, respectively. The pyridine (N1/C1–C4/C10) and phenyl (C12–C17) rings subtend a dihedral angle of 68.7 (2)° in mol­ecule *A* and 64.5 (2)° in mol­ecule *B*.

## 3. Supra­molecular features

**Figure 3:**
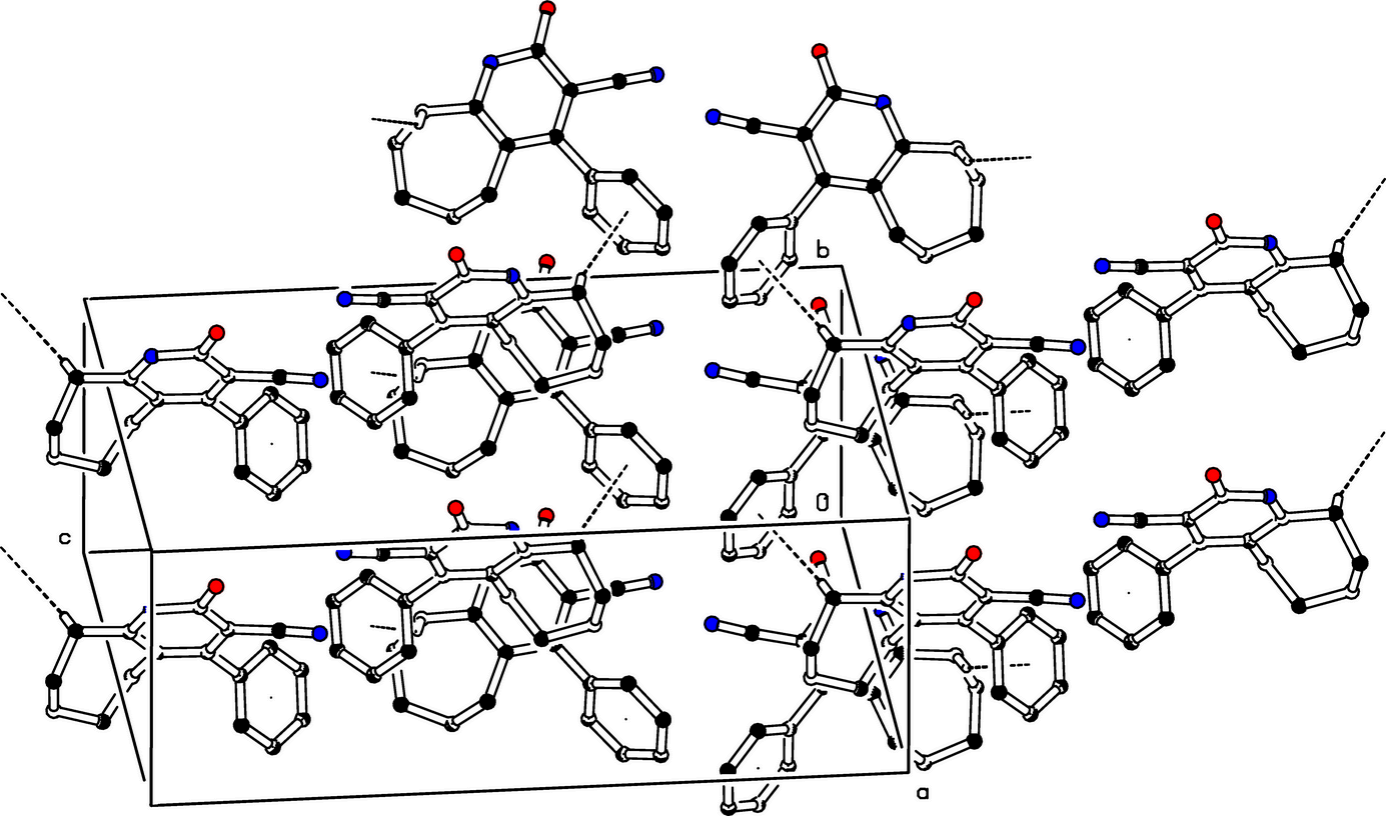
The crystal packing of (I). Inter­molecular C—H⋯π inter­actions are shown as dashed lines. For clarity, H atoms not involved in these inter­actions have been omitted.

The two mol­ecules in the asymmetric unit associate pairwise *via* N—H⋯O hydrogen bonds (Table 1 ▸) into dimers with an 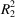
(8) graph-set motifs (Etter *et al.*, 1990 ▸; Bernstein *et al.*, 1995 ▸), as shown in Fig. 1 ▸. In the crystal, mol­ecules are further linked by weak C—H⋯π inter­actions, C9*A*—H9*A*1⋯*Cg*1 and C9*B*—H9*B*2⋯*Cg*2 (Table 1 ▸ and Fig. 3 ▸).

## 4. Hirshfeld surface analysis

**Figure 4:**
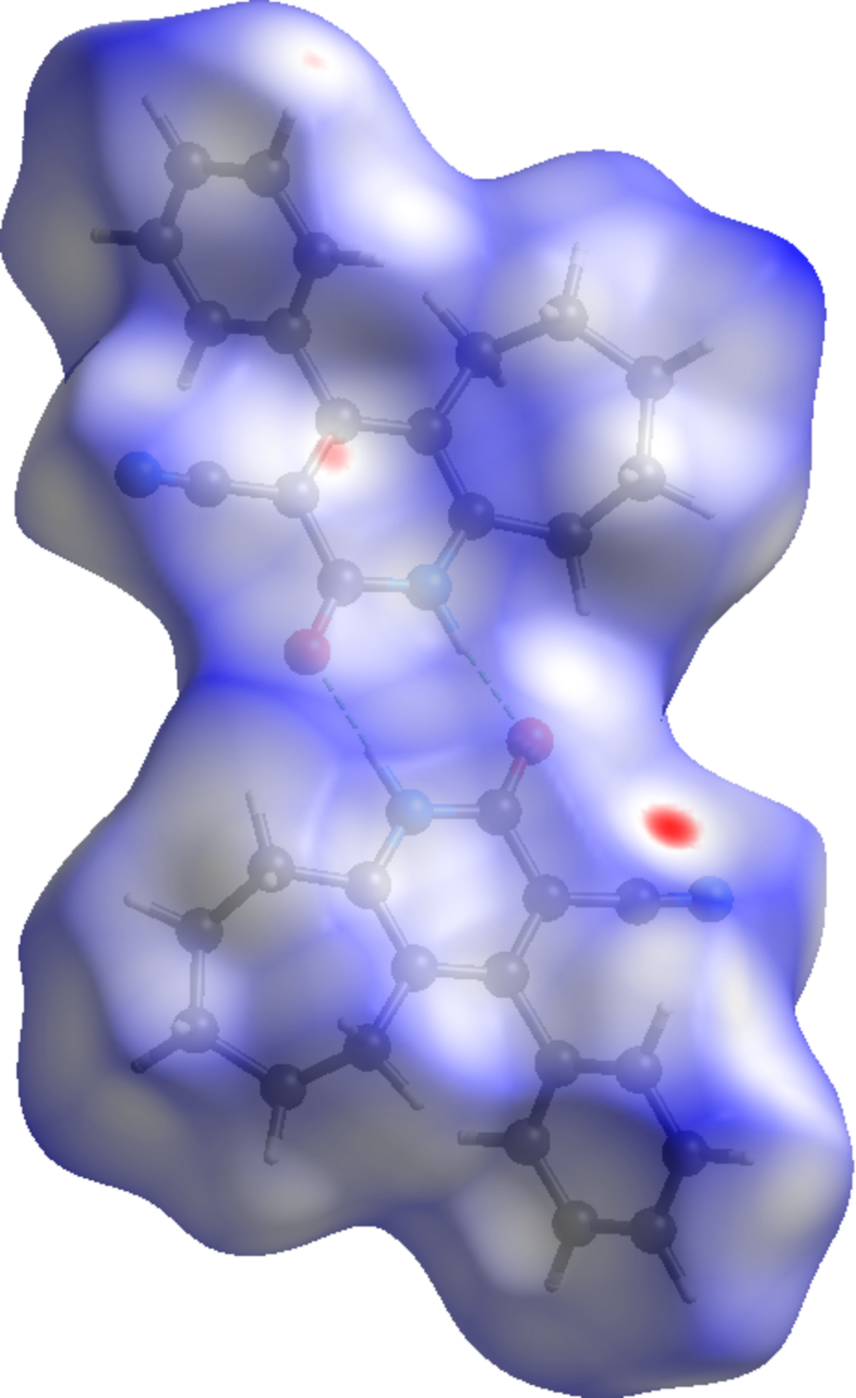
A view of the Hirshfeld surface mapped over *d*_norm_ for compound (I).

In order to further characterize and qu­antify the inter­molecular inter­actions in the title compound, a Hirshfeld surface (HS) analysis (Spackman & Jayatilaka, 2009 ▸) was carried out using *CrystalExplorer* (Spackman *et al.*, 2021 ▸). The HS mapped over *d*_norm_ is illustrated in Fig. 4 ▸.

**Figure 5:**
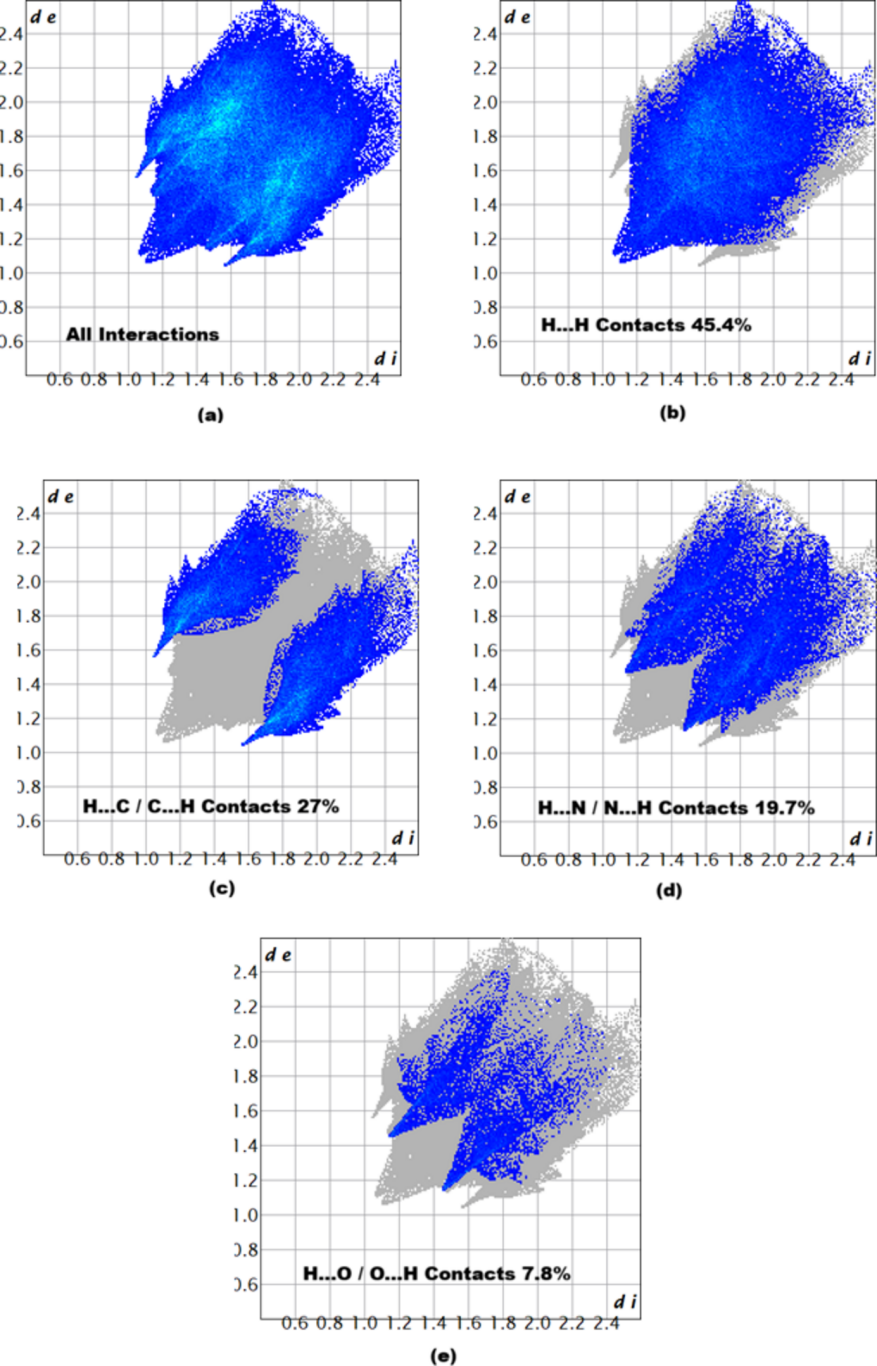
Two-dimensional fingerprint plots for compound (I), showing (*a*) all inter­actions, and delineated into (*b*) H⋯H, (*c*) H⋯C/C⋯H, (*d*) H⋯N/N⋯H, and (*e*) H⋯O/O⋯H inter­actions. The *d*_i_ and *d*_e_ values are the closest inter­nal and external distances (in Å) from given points on the Hirshfeld surface.

The associated two-dimensional fingerprint plots (McKinnon *et al.*, 2007 ▸) provide qu­anti­tative information about the non-covalent inter­actions in the crystal packing in terms of the percentage contribution of the inter­atomic contacts (Spackman & McKinnon, 2002 ▸). As shown in Fig. 5 ▸, the overall two-dimensional fingerprint plot for compound (I) is delineated into H⋯H, H⋯C/C⋯H, H⋯N/N⋯H and H⋯O/O⋯H contacts, revealing that H⋯H and H⋯C/C⋯H are the main contributors to the crystal packing.

## 5. Synthesis and crystallization

Compound (I) was synthesized by combining a mixture of benzaldehyde (1.0 ml), ethyl 2-cyano­acetate (1.1 ml) and cyclo­hepta­none (1.1 ml) with anhydrous ammonium acetate (0.7 g) in benzene (20 ml) as the reaction solvent. The resulting mixture was refluxed at 333 K for 4 h, and the progress of the reaction was monitored by thin-layer chromatography (TLC). After completion, the reaction mixture was cooled to room temperature, and the solvent was removed by evaporation under ambient conditions. The crude solid obtained was purified by recrystallization using a 1:1 (*v*/*v*) mixture of aceto­nitrile and ethanol, yielding a colourless crystalline compound of (I).

## 6. Refinement

Crystal data, data collection and structure refinement details are summarized in Table 2 ▸. All H atoms were placed in idealized positions and allowed to ride on their parent atoms: N—H = 0.86 Å and C—H = 0.93–0.97 Å with *U*_iso_(H) = 1.2*U*_eq_(C or N) for H atoms.

## Supplementary Material

Crystal structure: contains datablock(s) I, shelx. DOI: 10.1107/S2056989025010771/wm5777sup1.cif

Structure factors: contains datablock(s) I. DOI: 10.1107/S2056989025010771/wm5777Isup2.hkl

Supporting information file. DOI: 10.1107/S2056989025010771/wm5777Isup3.cml

CCDC reference: 2512805

Additional supporting information:  crystallographic information; 3D view; checkCIF report

## Figures and Tables

**Table 1:** Hydrogen-bond geometry (Å, °) *Cg*1 is the centroid of the phenyl ring in mol­ecule *B* (C12*B*–C17*B*) and *Cg*2 is the centroid of the phenyl ring in mol­ecule *A* (C12*A*–C17*A*).

*D*—H⋯*A*	*D*—H	H⋯*A*	*D*⋯*A*	*D*—H⋯*A*
N1*A*—H1*A*⋯O1*B*	0.86	1.91	2.751 (5)	165
N1*B*—H1*B*⋯O1*A*	0.86	1.96	2.808 (4)	170
C9*A*—H9*A*1⋯*Cg*1^i^	0.97	2.88	3.747 (5)	149
C9*B*—H9*B*2⋯*Cg*2^ii^	0.97	2.75	3.625 (5)	150

Symmetry codes: (i) 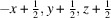; (ii) 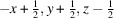.

**Table 2:** Experimental details

Crystal data	
Chemical formula	C_17_H_16_N_2_O
*M*_r_	264.32
Crystal system, space group	Orthorhombic, *Pna*2_1_
Temperature (K)	300
*a*, *b*, *c* (Å)	17.042 (2), 7.7479 (9), 21.166 (2)
*V* (Å^3^)	2794.7 (6)
*Z*	8
Radiation type	Mo *K*α
μ (mm^−1^)	0.08
Crystal size (mm)	0.36 × 0.22 × 0.09

Data collection	
Diffractometer	Bruker APEXII CCD
Absorption correction	Multi-scan (*SADABS*; Krause *et al.*, 2015 ▸)
*T*_min_, *T*_max_	0.576, 0.746
No. of measured, independent and observed [*I* > 2σ(*I*)] reflections	25904, 6466, 4063
*R*_int_	0.045
(sin θ/λ)_max_ (Å^−1^)	0.667

Refinement	
*R*[*F*^2^ > 2σ(*F*^2^)], *wR*(*F*^2^), *S*	0.053, 0.175, 1.10
No. of reflections	6466
No. of parameters	362
No. of restraints	1
H-atom treatment	H-atom parameters constrained
Δρ_max_, Δρ_min_ (e Å^−3^)	0.25, −0.21
Absolute structure	Flack *x* determined using 1477 quotients [(*I*^+^)−(*I*^−^)]/[(*I*^+^)+(*I*^−^)] (Parsons *et al.*, 2013 ▸)
Absolute structure parameter	0.3 (7)

Computer programs: *APEX3* and *SAINT* (Bruker, 2017 ▸), *SHELXT* (Sheldrick, 2015*a* ▸), *ORTEP-3 for Windows* (Farrugia, 2012 ▸), *SHELXL* (Sheldrick, 2015*b* ▸) and *PLATON* (Spek, 2020 ▸).

## supplementary crystallographic information

### 2-Oxo-4-phenyl-2,5,6,7,8,9-hexahydro-1H-cyclohepta[b]pyridine-3-carbonitrile Crystal data

**Table:**

C_17_H_16_N_2_O	*D*_x_ = 1.256 Mg m^−^^3^
*M**_r_* = 264.32	Mo *K*α radiation, λ = 0.71073 Å
Orthorhombic, *P**n**a*2_1_	Cell parameters from 9985 reflections
*a* = 17.042 (2) Å	θ = 2.4–25.9°
*b* = 7.7479 (9) Å	µ = 0.08 mm^−^^1^
*c* = 21.166 (2) Å	*T* = 300 K
*V* = 2794.7 (6) Å^3^	Block, colourless
*Z* = 8	0.36 × 0.22 × 0.09 mm
*F*(000) = 1120	

### 2-Oxo-4-phenyl-2,5,6,7,8,9-hexahydro-1H-cyclohepta[b]pyridine-3-carbonitrile Data collection

**Table:**

Bruker APEXII CCD diffractometer	4063 reflections with *I* > 2σ(*I*)
Radiation source: i-mu-s microfocus source	*R*_int_ = 0.045
φ and ω scans	θ_max_ = 28.3°, θ_min_ = 1.9°
Absorption correction: multi-scan (*SADABS*; Krause *et al.*, 2015)	*h* = −16→22
*T*_min_ = 0.576, *T*_max_ = 0.746	*k* = −10→10
25904 measured reflections	*l* = −23→28
6466 independent reflections	

### 2-Oxo-4-phenyl-2,5,6,7,8,9-hexahydro-1H-cyclohepta[b]pyridine-3-carbonitrile Refinement

**Table:**

Refinement on *F*^2^	H-atom parameters constrained
Least-squares matrix: full	*w* = 1/[σ^2^(*F*_o_^2^) + (0.0771*P*)^2^ + 0.7116*P*] where *P* = (*F*_o_^2^ + 2*F*_c_^2^)/3
*R*[*F*^2^ > 2σ(*F*^2^)] = 0.053	(Δ/σ)_max_ < 0.001
*wR*(*F*^2^) = 0.175	Δρ_max_ = 0.25 e Å^−^^3^
*S* = 1.10	Δρ_min_ = −0.21 e Å^−^^3^
6466 reflections	Extinction correction: SHELXL2019/2 (Sheldrick 2015b), Fc^*^=kFc[1+0.001xFc^2^λ^3^/sin(2θ)]^-1/4^
362 parameters	Extinction coefficient: 0.0079 (16)
1 restraint	Absolute structure: Flack *x* determined using 1477 quotients [(*I*^+^)-(*I*^-^)]/[(*I*^+^)+(*I*^-^)] (Parsons *et al.*, 2013)
Hydrogen site location: inferred from neighbouring sites	Absolute structure parameter: 0.3 (7)

### 2-Oxo-4-phenyl-2,5,6,7,8,9-hexahydro-1H-cyclohepta[b]pyridine-3-carbonitrile Special details

**Table:**

Geometry. All esds (except the esd in the dihedral angle between two l.s. planes) are estimated using the full covariance matrix. The cell esds are taken into account individually in the estimation of esds in distances, angles and torsion angles; correlations between esds in cell parameters are only used when they are defined by crystal symmetry. An approximate (isotropic) treatment of cell esds is used for estimating esds involving l.s. planes.

### 2-Oxo-4-phenyl-2,5,6,7,8,9-hexahydro-1H-cyclohepta[b]pyridine-3-carbonitrile Fractional atomic coordinates and isotropic or equivalent isotropic displacement parameters (Å^2^)

**Table:**

	*x*	*y*	*z*	*U*_iso_*/*U*_eq_	
O1A	0.39241 (19)	0.5430 (4)	0.37967 (14)	0.0581 (8)	
N1A	0.4532 (2)	0.6852 (5)	0.45990 (16)	0.0483 (9)	
H1A	0.434021	0.611161	0.485950	0.058*	
N2A	0.4296 (3)	0.7840 (7)	0.2394 (3)	0.0819 (14)	
C1A	0.4356 (3)	0.6644 (5)	0.3969 (2)	0.0457 (10)	
C2A	0.4698 (3)	0.7918 (6)	0.35642 (19)	0.0454 (10)	
C3A	0.5166 (2)	0.9248 (5)	0.37957 (19)	0.0421 (9)	
C4A	0.5319 (2)	0.9352 (5)	0.44519 (19)	0.0448 (9)	
C5A	0.5818 (2)	1.0749 (6)	0.4735 (2)	0.0521 (11)	
H5A1	0.606335	1.139354	0.439545	0.062*	
H5A2	0.623189	1.021728	0.498145	0.062*	
C6A	0.5364 (3)	1.2018 (6)	0.5161 (2)	0.0602 (12)	
H6A1	0.566405	1.307821	0.519563	0.072*	
H6A2	0.486908	1.229724	0.495925	0.072*	
C7A	0.5200 (3)	1.1338 (7)	0.5820 (2)	0.0699 (15)	
H7A1	0.569907	1.113736	0.602807	0.084*	
H7A2	0.493027	1.223363	0.605544	0.084*	
C8A	0.4719 (3)	0.9697 (7)	0.5867 (2)	0.0649 (13)	
H8A1	0.420624	0.991530	0.568446	0.078*	
H8A2	0.464215	0.942871	0.630988	0.078*	
C9A	0.5070 (3)	0.8123 (6)	0.5544 (2)	0.0551 (11)	
H9A1	0.562400	0.806080	0.564699	0.066*	
H9A2	0.482056	0.709541	0.571129	0.066*	
C10A	0.4982 (2)	0.8129 (6)	0.4841 (2)	0.0445 (9)	
C11A	0.4487 (3)	0.7852 (6)	0.2908 (2)	0.0533 (11)	
C12A	0.5474 (2)	1.0555 (5)	0.33410 (19)	0.0433 (9)	
C13A	0.6034 (3)	1.0073 (6)	0.2894 (2)	0.0521 (11)	
H13A	0.622612	0.894911	0.288708	0.062*	
C14A	0.6303 (3)	1.1277 (6)	0.2462 (2)	0.0608 (12)	
H14A	0.667972	1.095544	0.216655	0.073*	
C15A	0.6022 (3)	1.2938 (7)	0.2463 (3)	0.0640 (13)	
H15A	0.620495	1.373770	0.217027	0.077*	
C16A	0.5461 (3)	1.3407 (6)	0.2905 (3)	0.0656 (13)	
H16A	0.526451	1.452663	0.290765	0.079*	
C17A	0.5194 (3)	1.2228 (6)	0.3341 (3)	0.0574 (12)	
H17A	0.482207	1.255955	0.363877	0.069*	
O1B	0.36828 (19)	0.4830 (4)	0.54204 (15)	0.0599 (8)	
N1B	0.3092 (2)	0.3297 (5)	0.46348 (16)	0.0472 (8)	
H1B	0.328985	0.399858	0.436319	0.057*	
N2B	0.3269 (3)	0.2852 (6)	0.6855 (2)	0.0765 (13)	
C1B	0.3258 (2)	0.3588 (5)	0.5261 (2)	0.0475 (10)	
C2B	0.2919 (3)	0.2359 (5)	0.5686 (2)	0.0457 (10)	
C3B	0.2470 (2)	0.0979 (5)	0.54742 (19)	0.0430 (9)	
C4B	0.2312 (2)	0.0797 (6)	0.48201 (19)	0.0430 (9)	
C5B	0.1800 (3)	−0.0623 (6)	0.4559 (2)	0.0529 (11)	
H5B1	0.138381	−0.010494	0.431058	0.064*	
H5B2	0.155759	−0.122991	0.490877	0.064*	
C6B	0.2233 (3)	−0.1933 (6)	0.4147 (2)	0.0603 (12)	
H6B1	0.273513	−0.219164	0.434107	0.072*	
H6B2	0.193122	−0.299359	0.413494	0.072*	
C7B	0.2373 (3)	−0.1318 (7)	0.3472 (2)	0.0659 (13)	
H7B1	0.262198	−0.224510	0.323815	0.079*	
H7B2	0.186828	−0.109808	0.327653	0.079*	
C8B	0.2875 (3)	0.0294 (7)	0.3406 (2)	0.0663 (13)	
H8B1	0.296098	0.051284	0.295979	0.080*	
H8B2	0.338295	0.007270	0.359633	0.080*	
C9B	0.2526 (3)	0.1936 (6)	0.3708 (2)	0.0529 (11)	
H9B1	0.277171	0.294396	0.352191	0.063*	
H9B2	0.196876	0.198874	0.361486	0.063*	
C10B	0.2640 (2)	0.1982 (5)	0.4410 (2)	0.0448 (10)	
C11B	0.3104 (3)	0.2590 (6)	0.6339 (2)	0.0543 (11)	
C12B	0.2161 (2)	−0.0299 (5)	0.5940 (2)	0.0445 (9)	
C13B	0.1631 (3)	0.0195 (6)	0.6395 (2)	0.0554 (11)	
H13B	0.147220	0.134158	0.642202	0.066*	
C14B	0.1332 (3)	−0.1015 (7)	0.6814 (2)	0.0686 (14)	
H14B	0.096692	−0.068033	0.711717	0.082*	
C15B	0.1572 (3)	−0.2703 (7)	0.6785 (3)	0.0690 (15)	
H15B	0.136028	−0.351439	0.706010	0.083*	
C16B	0.2120 (3)	−0.3191 (6)	0.6352 (3)	0.0644 (13)	
H16B	0.229811	−0.432519	0.634532	0.077*	
C17B	0.2413 (3)	−0.2009 (6)	0.5923 (2)	0.0549 (11)	
H17B	0.277839	−0.235620	0.562247	0.066*	

### 2-Oxo-4-phenyl-2,5,6,7,8,9-hexahydro-1H-cyclohepta[b]pyridine-3-carbonitrile Atomic displacement parameters (Å^2^)

**Table:**

	*U*^11^	*U*^22^	*U*^33^	*U*^12^	*U*^13^	*U*^23^
O1A	0.0721 (19)	0.0524 (18)	0.0499 (18)	−0.0166 (16)	0.0009 (16)	−0.0055 (14)
N1A	0.056 (2)	0.048 (2)	0.041 (2)	−0.0075 (17)	0.0043 (17)	0.0058 (16)
N2A	0.111 (4)	0.089 (3)	0.046 (3)	−0.030 (3)	−0.007 (3)	0.000 (2)
C1A	0.054 (2)	0.041 (2)	0.042 (2)	−0.0004 (19)	0.0027 (19)	−0.0010 (18)
C2A	0.056 (2)	0.045 (2)	0.035 (2)	−0.0013 (19)	0.0013 (18)	0.0022 (17)
C3A	0.044 (2)	0.044 (2)	0.038 (2)	0.0005 (17)	0.0041 (17)	−0.0017 (17)
C4A	0.045 (2)	0.048 (2)	0.041 (2)	−0.0027 (18)	0.0020 (17)	−0.0027 (18)
C5A	0.051 (2)	0.063 (3)	0.043 (2)	−0.013 (2)	0.0023 (19)	−0.005 (2)
C6A	0.068 (3)	0.060 (3)	0.053 (3)	−0.011 (2)	0.003 (2)	−0.009 (2)
C7A	0.083 (4)	0.075 (4)	0.052 (3)	−0.009 (3)	0.010 (3)	−0.017 (3)
C8A	0.073 (3)	0.078 (3)	0.044 (3)	−0.012 (3)	0.012 (2)	−0.003 (2)
C9A	0.060 (3)	0.062 (3)	0.043 (3)	−0.008 (2)	−0.004 (2)	0.006 (2)
C10A	0.043 (2)	0.050 (2)	0.041 (2)	−0.0014 (18)	−0.0007 (17)	0.000 (2)
C11A	0.066 (3)	0.050 (3)	0.044 (3)	−0.008 (2)	−0.002 (2)	−0.004 (2)
C12A	0.050 (2)	0.043 (2)	0.037 (2)	−0.0033 (18)	−0.0019 (17)	0.0003 (17)
C13A	0.065 (3)	0.048 (2)	0.043 (2)	−0.001 (2)	0.005 (2)	−0.003 (2)
C14A	0.072 (3)	0.064 (3)	0.047 (3)	−0.010 (2)	0.008 (2)	0.001 (2)
C15A	0.083 (3)	0.061 (3)	0.048 (3)	−0.017 (3)	−0.006 (3)	0.014 (2)
C16A	0.077 (3)	0.048 (3)	0.072 (3)	0.001 (2)	−0.013 (3)	0.007 (3)
C17A	0.059 (3)	0.048 (2)	0.066 (3)	0.002 (2)	0.002 (2)	−0.002 (2)
O1B	0.075 (2)	0.0540 (18)	0.0507 (18)	−0.0184 (16)	0.0028 (16)	−0.0007 (14)
N1B	0.0525 (19)	0.046 (2)	0.043 (2)	−0.0052 (16)	0.0027 (16)	0.0059 (15)
N2B	0.115 (4)	0.063 (3)	0.052 (3)	−0.004 (3)	−0.012 (3)	−0.001 (2)
C1B	0.053 (2)	0.046 (2)	0.044 (2)	−0.0027 (19)	0.0046 (19)	0.0001 (19)
C2B	0.053 (2)	0.044 (2)	0.040 (2)	−0.0006 (19)	0.0015 (18)	0.0008 (18)
C3B	0.045 (2)	0.044 (2)	0.040 (2)	0.0040 (17)	0.0042 (18)	0.0022 (17)
C4B	0.045 (2)	0.044 (2)	0.040 (2)	0.0016 (17)	0.0061 (17)	0.0021 (18)
C5B	0.054 (2)	0.059 (3)	0.045 (2)	−0.009 (2)	0.0003 (19)	0.002 (2)
C6B	0.071 (3)	0.053 (3)	0.057 (3)	−0.006 (2)	0.003 (2)	−0.005 (2)
C7B	0.077 (3)	0.067 (3)	0.054 (3)	−0.005 (3)	0.006 (2)	−0.012 (2)
C8B	0.079 (3)	0.073 (3)	0.047 (3)	−0.007 (3)	0.016 (2)	−0.006 (2)
C9B	0.060 (3)	0.059 (3)	0.040 (3)	−0.008 (2)	−0.002 (2)	0.007 (2)
C10B	0.045 (2)	0.045 (2)	0.045 (3)	0.0019 (18)	0.0032 (17)	0.0017 (19)
C11B	0.073 (3)	0.046 (2)	0.044 (3)	0.001 (2)	0.000 (2)	0.0019 (19)
C12B	0.053 (2)	0.040 (2)	0.041 (2)	−0.0012 (18)	0.0015 (18)	0.0025 (18)
C13B	0.068 (3)	0.052 (2)	0.047 (3)	0.003 (2)	0.013 (2)	0.008 (2)
C14B	0.081 (3)	0.078 (4)	0.047 (3)	−0.007 (3)	0.016 (2)	0.010 (3)
C15B	0.094 (4)	0.064 (3)	0.049 (3)	−0.019 (3)	−0.014 (3)	0.022 (2)
C16B	0.082 (3)	0.046 (3)	0.065 (3)	−0.002 (2)	−0.013 (3)	0.011 (2)
C17B	0.060 (3)	0.048 (2)	0.057 (3)	0.009 (2)	−0.001 (2)	−0.001 (2)

### 2-Oxo-4-phenyl-2,5,6,7,8,9-hexahydro-1H-cyclohepta[b]pyridine-3-carbonitrile Geometric parameters (Å, º)

**Table:**

O1A—C1A	1.248 (5)	O1B—C1B	1.251 (5)
N1A—C10A	1.353 (6)	N1B—C10B	1.363 (5)
N1A—C1A	1.377 (5)	N1B—C1B	1.373 (6)
N1A—H1A	0.8600	N1B—H1B	0.8600
N2A—C11A	1.135 (7)	N2B—C11B	1.145 (6)
C1A—C2A	1.431 (6)	C1B—C2B	1.432 (6)
C2A—C3A	1.393 (6)	C2B—C3B	1.388 (6)
C2A—C11A	1.436 (7)	C2B—C11B	1.429 (7)
C3A—C4A	1.415 (6)	C3B—C4B	1.417 (6)
C3A—C12A	1.492 (6)	C3B—C12B	1.493 (6)
C4A—C10A	1.381 (6)	C4B—C10B	1.381 (6)
C4A—C5A	1.500 (6)	C4B—C5B	1.510 (6)
C5A—C6A	1.542 (7)	C5B—C6B	1.527 (7)
C5A—H5A1	0.9700	C5B—H5B1	0.9700
C5A—H5A2	0.9700	C5B—H5B2	0.9700
C6A—C7A	1.518 (7)	C6B—C7B	1.525 (7)
C6A—H6A1	0.9700	C6B—H6B1	0.9700
C6A—H6A2	0.9700	C6B—H6B2	0.9700
C7A—C8A	1.515 (7)	C7B—C8B	1.521 (7)
C7A—H7A1	0.9700	C7B—H7B1	0.9700
C7A—H7A2	0.9700	C7B—H7B2	0.9700
C8A—C9A	1.520 (7)	C8B—C9B	1.544 (7)
C8A—H8A1	0.9700	C8B—H8B1	0.9700
C8A—H8A2	0.9700	C8B—H8B2	0.9700
C9A—C10A	1.495 (6)	C9B—C10B	1.499 (6)
C9A—H9A1	0.9700	C9B—H9B1	0.9700
C9A—H9A2	0.9700	C9B—H9B2	0.9700
C12A—C17A	1.381 (6)	C12B—C13B	1.376 (6)
C12A—C13A	1.394 (6)	C12B—C17B	1.393 (6)
C13A—C14A	1.385 (6)	C13B—C14B	1.388 (6)
C13A—H13A	0.9300	C13B—H13B	0.9300
C14A—C15A	1.374 (7)	C14B—C15B	1.372 (7)
C14A—H14A	0.9300	C14B—H14B	0.9300
C15A—C16A	1.386 (8)	C15B—C16B	1.361 (8)
C15A—H15A	0.9300	C15B—H15B	0.9300
C16A—C17A	1.376 (7)	C16B—C17B	1.384 (7)
C16A—H16A	0.9300	C16B—H16B	0.9300
C17A—H17A	0.9300	C17B—H17B	0.9300
			
C10A—N1A—C1A	125.2 (4)	C10B—N1B—C1B	125.1 (4)
C10A—N1A—H1A	117.4	C10B—N1B—H1B	117.4
C1A—N1A—H1A	117.4	C1B—N1B—H1B	117.4
O1A—C1A—N1A	120.0 (4)	O1B—C1B—N1B	120.4 (4)
O1A—C1A—C2A	125.8 (4)	O1B—C1B—C2B	125.1 (4)
N1A—C1A—C2A	114.2 (4)	N1B—C1B—C2B	114.5 (4)
C3A—C2A—C1A	122.3 (4)	C3B—C2B—C11B	122.1 (4)
C3A—C2A—C11A	120.7 (4)	C3B—C2B—C1B	122.1 (4)
C1A—C2A—C11A	116.9 (4)	C11B—C2B—C1B	115.8 (4)
C2A—C3A—C4A	119.5 (4)	C2B—C3B—C4B	119.8 (4)
C2A—C3A—C12A	118.5 (4)	C2B—C3B—C12B	119.5 (4)
C4A—C3A—C12A	122.0 (4)	C4B—C3B—C12B	120.8 (4)
C10A—C4A—C3A	118.0 (4)	C10B—C4B—C3B	118.1 (4)
C10A—C4A—C5A	119.5 (4)	C10B—C4B—C5B	119.2 (4)
C3A—C4A—C5A	122.5 (4)	C3B—C4B—C5B	122.7 (4)
C4A—C5A—C6A	114.2 (3)	C4B—C5B—C6B	114.5 (4)
C4A—C5A—H5A1	108.7	C4B—C5B—H5B1	108.6
C6A—C5A—H5A1	108.7	C6B—C5B—H5B1	108.6
C4A—C5A—H5A2	108.7	C4B—C5B—H5B2	108.6
C6A—C5A—H5A2	108.7	C6B—C5B—H5B2	108.6
H5A1—C5A—H5A2	107.6	H5B1—C5B—H5B2	107.6
C7A—C6A—C5A	114.1 (4)	C7B—C6B—C5B	113.7 (4)
C7A—C6A—H6A1	108.7	C7B—C6B—H6B1	108.8
C5A—C6A—H6A1	108.7	C5B—C6B—H6B1	108.8
C7A—C6A—H6A2	108.7	C7B—C6B—H6B2	108.8
C5A—C6A—H6A2	108.7	C5B—C6B—H6B2	108.8
H6A1—C6A—H6A2	107.6	H6B1—C6B—H6B2	107.7
C8A—C7A—C6A	116.8 (4)	C8B—C7B—C6B	115.5 (4)
C8A—C7A—H7A1	108.1	C8B—C7B—H7B1	108.4
C6A—C7A—H7A1	108.1	C6B—C7B—H7B1	108.4
C8A—C7A—H7A2	108.1	C8B—C7B—H7B2	108.4
C6A—C7A—H7A2	108.1	C6B—C7B—H7B2	108.4
H7A1—C7A—H7A2	107.3	H7B1—C7B—H7B2	107.5
C7A—C8A—C9A	115.5 (4)	C7B—C8B—C9B	114.9 (4)
C7A—C8A—H8A1	108.4	C7B—C8B—H8B1	108.5
C9A—C8A—H8A1	108.4	C9B—C8B—H8B1	108.5
C7A—C8A—H8A2	108.4	C7B—C8B—H8B2	108.5
C9A—C8A—H8A2	108.4	C9B—C8B—H8B2	108.5
H8A1—C8A—H8A2	107.5	H8B1—C8B—H8B2	107.5
C10A—C9A—C8A	113.9 (4)	C10B—C9B—C8B	112.4 (4)
C10A—C9A—H9A1	108.8	C10B—C9B—H9B1	109.1
C8A—C9A—H9A1	108.8	C8B—C9B—H9B1	109.1
C10A—C9A—H9A2	108.8	C10B—C9B—H9B2	109.1
C8A—C9A—H9A2	108.8	C8B—C9B—H9B2	109.1
H9A1—C9A—H9A2	107.7	H9B1—C9B—H9B2	107.9
N1A—C10A—C4A	120.8 (4)	N1B—C10B—C4B	120.4 (4)
N1A—C10A—C9A	115.6 (4)	N1B—C10B—C9B	115.9 (4)
C4A—C10A—C9A	123.6 (4)	C4B—C10B—C9B	123.7 (4)
N2A—C11A—C2A	177.4 (6)	N2B—C11B—C2B	176.6 (5)
C17A—C12A—C13A	119.3 (4)	C13B—C12B—C17B	119.0 (4)
C17A—C12A—C3A	121.0 (4)	C13B—C12B—C3B	120.6 (4)
C13A—C12A—C3A	119.7 (4)	C17B—C12B—C3B	120.4 (4)
C14A—C13A—C12A	119.6 (4)	C12B—C13B—C14B	120.0 (4)
C14A—C13A—H13A	120.2	C12B—C13B—H13B	120.0
C12A—C13A—H13A	120.2	C14B—C13B—H13B	120.0
C15A—C14A—C13A	121.0 (5)	C15B—C14B—C13B	120.4 (5)
C15A—C14A—H14A	119.5	C15B—C14B—H14B	119.8
C13A—C14A—H14A	119.5	C13B—C14B—H14B	119.8
C14A—C15A—C16A	119.2 (5)	C16B—C15B—C14B	120.0 (5)
C14A—C15A—H15A	120.4	C16B—C15B—H15B	120.0
C16A—C15A—H15A	120.4	C14B—C15B—H15B	120.0
C17A—C16A—C15A	120.4 (5)	C15B—C16B—C17B	120.4 (5)
C17A—C16A—H16A	119.8	C15B—C16B—H16B	119.8
C15A—C16A—H16A	119.8	C17B—C16B—H16B	119.8
C16A—C17A—C12A	120.6 (5)	C16B—C17B—C12B	120.1 (5)
C16A—C17A—H17A	119.7	C16B—C17B—H17B	119.9
C12A—C17A—H17A	119.7	C12B—C17B—H17B	119.9
			
C10A—N1A—C1A—O1A	−179.0 (4)	C10B—N1B—C1B—O1B	179.8 (4)
C10A—N1A—C1A—C2A	0.3 (6)	C10B—N1B—C1B—C2B	0.7 (6)
O1A—C1A—C2A—C3A	179.1 (4)	O1B—C1B—C2B—C3B	−178.1 (4)
N1A—C1A—C2A—C3A	−0.1 (6)	N1B—C1B—C2B—C3B	0.9 (6)
O1A—C1A—C2A—C11A	4.0 (7)	O1B—C1B—C2B—C11B	−1.2 (7)
N1A—C1A—C2A—C11A	−175.3 (4)	N1B—C1B—C2B—C11B	177.8 (4)
C1A—C2A—C3A—C4A	0.6 (6)	C11B—C2B—C3B—C4B	−179.1 (4)
C11A—C2A—C3A—C4A	175.5 (4)	C1B—C2B—C3B—C4B	−2.4 (6)
C1A—C2A—C3A—C12A	−177.8 (4)	C11B—C2B—C3B—C12B	0.7 (6)
C11A—C2A—C3A—C12A	−2.9 (6)	C1B—C2B—C3B—C12B	177.4 (4)
C2A—C3A—C4A—C10A	−1.1 (6)	C2B—C3B—C4B—C10B	2.3 (6)
C12A—C3A—C4A—C10A	177.2 (4)	C12B—C3B—C4B—C10B	−177.5 (4)
C2A—C3A—C4A—C5A	−180.0 (4)	C2B—C3B—C4B—C5B	−177.2 (4)
C12A—C3A—C4A—C5A	−1.7 (6)	C12B—C3B—C4B—C5B	2.9 (6)
C10A—C4A—C5A—C6A	−66.1 (6)	C10B—C4B—C5B—C6B	66.8 (5)
C3A—C4A—C5A—C6A	112.7 (5)	C3B—C4B—C5B—C6B	−113.7 (5)
C4A—C5A—C6A—C7A	79.2 (5)	C4B—C5B—C6B—C7B	−80.2 (5)
C5A—C6A—C7A—C8A	−59.8 (6)	C5B—C6B—C7B—C8B	61.4 (6)
C6A—C7A—C8A—C9A	60.1 (7)	C6B—C7B—C8B—C9B	−62.8 (6)
C7A—C8A—C9A—C10A	−76.9 (6)	C7B—C8B—C9B—C10B	79.0 (5)
C1A—N1A—C10A—C4A	−0.9 (6)	C1B—N1B—C10B—C4B	−0.8 (6)
C1A—N1A—C10A—C9A	178.1 (4)	C1B—N1B—C10B—C9B	178.8 (4)
C3A—C4A—C10A—N1A	1.3 (6)	C3B—C4B—C10B—N1B	−0.8 (6)
C5A—C4A—C10A—N1A	−179.8 (4)	C5B—C4B—C10B—N1B	178.8 (4)
C3A—C4A—C10A—C9A	−177.7 (4)	C3B—C4B—C10B—C9B	179.6 (4)
C5A—C4A—C10A—C9A	1.2 (6)	C5B—C4B—C10B—C9B	−0.8 (6)
C8A—C9A—C10A—N1A	−115.8 (5)	C8B—C9B—C10B—N1B	116.8 (4)
C8A—C9A—C10A—C4A	63.3 (6)	C8B—C9B—C10B—C4B	−63.6 (6)
C2A—C3A—C12A—C17A	109.5 (5)	C2B—C3B—C12B—C13B	63.9 (5)
C4A—C3A—C12A—C17A	−68.8 (6)	C4B—C3B—C12B—C13B	−116.2 (5)
C2A—C3A—C12A—C13A	−68.4 (5)	C2B—C3B—C12B—C17B	−115.4 (5)
C4A—C3A—C12A—C13A	113.3 (5)	C4B—C3B—C12B—C17B	64.4 (6)
C17A—C12A—C13A—C14A	0.4 (7)	C17B—C12B—C13B—C14B	−2.4 (7)
C3A—C12A—C13A—C14A	178.2 (4)	C3B—C12B—C13B—C14B	178.3 (4)
C12A—C13A—C14A—C15A	−0.5 (7)	C12B—C13B—C14B—C15B	1.1 (8)
C13A—C14A—C15A—C16A	0.1 (8)	C13B—C14B—C15B—C16B	1.6 (8)
C14A—C15A—C16A—C17A	0.5 (8)	C14B—C15B—C16B—C17B	−2.8 (8)
C15A—C16A—C17A—C12A	−0.6 (8)	C15B—C16B—C17B—C12B	1.4 (7)
C13A—C12A—C17A—C16A	0.2 (7)	C13B—C12B—C17B—C16B	1.2 (7)
C3A—C12A—C17A—C16A	−177.6 (4)	C3B—C12B—C17B—C16B	−179.5 (4)

### 2-Oxo-4-phenyl-2,5,6,7,8,9-hexahydro-1H-cyclohepta[b]pyridine-3-carbonitrile Hydrogen-bond geometry (Å, º)

*Cg*1 is the centroid of the phenyl ring in molecule *B* (C12*B*–C17*B*) and *Cg*2 is the centroid of the phenyl ring in molecule *A* (C12*A*–C17*A*).

**Table:**

*D*—H···*A*	*D*—H	H···*A*	*D*···*A*	*D*—H···*A*
N1*A*—H1*A*···O1*B*	0.86	1.91	2.751 (5)	165
N1*B*—H1*B*···O1*A*	0.86	1.96	2.808 (4)	170
C9*A*—H9*A*1···*Cg*1^i^	0.97	2.88	3.747 (5)	149
C9*B*—H9*B*2···*Cg*2^ii^	0.97	2.75	3.625 (5)	150

Symmetry codes: (i) −*x*+1/2, *y*+1/2, *z*+1/2; (ii) −*x*+1/2, *y*+1/2, *z*−1/2.
